# Debriefing interaction patterns and learning outcomes in simulation: an observational mixed-methods network study

**DOI:** 10.1186/s41077-022-00222-3

**Published:** 2022-09-06

**Authors:** Sandra Abegglen, Robert Greif, Yves Balmer, Hans Joerg Znoj, Sabine Nabecker

**Affiliations:** 1 Department of Health Psychology and Behavioural Medicine, University of Bern, 3012 Bern, Switzerland; 2 Department of Anaesthesiology and Pain Medicine, Inselspital, Bern University Hospital, University of Bern, Bern, Switzerland; 3 School of Medicine, Sigmund Freud University Vienna, Vienna, Austria; 4 Department of Anaesthesiology and Pain Management, Sinai Health System, University of Toronto, Toronto, Canada

## Abstract

**Background:**

Debriefing is effective and inexpensive to increase learning benefits of participants in simulation-based medical education. However, suitable communication patterns during debriefings remain to be defined. This study aimed to explore interaction patterns during debriefings and to link these to participants’ satisfaction, perceived usefulness, and self-reported learning outcomes.

**Methods:**

We assessed interaction patterns during debriefings of simulation sessions for residents, specialists, and nurses from the local anaesthesia department at the Bern University Hospital, Bern, Switzerland. Network analysis was applied to establish distinctive interaction pattern categories based on recorded interaction links. We used multilevel modelling to assess relationships between interaction patterns and self-reported learning outcomes.

**Results:**

Out of 57 debriefings that involved 111 participants, discriminatory analyses revealed three distinctive interaction patterns: ‘fan’, ‘triangle’, and ‘net’. Participants reported significantly higher self-reported learning effects in debriefings with a net pattern, compared to debriefings with a fan pattern. No effects were observed for participant satisfaction, learning effects after 1 month, and perceived usefulness of simulation sessions.

**Conclusions:**

A learner-centred interaction pattern (i.e. net) was significantly associated with improved short-term self-reported individual learning and team learning. This supports good-practice debriefing guidelines, which stated that participants should have a high activity in debriefings, guided by debriefers, who facilitate discussions to maximize the development for the learners.

**Supplementary Information:**

The online version contains supplementary material available at 10.1186/s41077-022-00222-3.

## Introduction

Simulation-based medical education enables mastery learning through deliberate practice of high-risk events without endangering patients. It is effective for learning of individuals [1, 2], changes in team behaviours, and it also improves patient outcomes [3–5]. However, despite widespread implementation of simulations at all levels of medical education, the advantages of different simulation approaches remain to be defined [4]. This calls for more ‘fine-grained’ simulation research that is focused on debriefing, one core component of simulation, and factors influencing its effectiveness [5–8].

Debriefings are ‘after-action reviews’[8] aiming to change behaviour and learning. Current literature suggests that debriefings are the pivotal way to maximize individual learning processes and thus facilitate behavioural changes at the level of individuals, teams, and systems [6–10]. The facilitator of this process, the debriefer, should primarily moderate group discussions and stimulate learning [6, 8, 9, 11–13]. Dieckmann and collegues [6] observed that debriefers still were the most active persons in debriefings, often engaged in a dyadic communication pattern with the most active participants. Although experienced simulation instructors are trained in various debriefing approaches, there is little evidence how different interaction patterns during debriefing influence learning of individuals and groups. Ideal interaction patterns were defined as balanced interactions connecting participants with each other and with the debriefer [6].

Kolbe and Boos [14] recommended for the study of debriefing effectiveness a focus on group dynamics. This allows opening the black box of the group process as a true mediator between debriefers’ behaviour and ultimately learning. Therefore, investigating different interaction patterns (i.e. debriefing styles) is of utmost importance. Linking these patterns to learning outcomes finally might establish desirable changes in behaviour.

To date, most studies have focused only on the individual effects of debriefer behaviors [6, 15]. Social network analysis is a well-established method to analyse interaction patterns also at the group and system level [16]. Although it is rarely used in medical education research [14, 17], in the field of small-group research, social network analysis has already been shown to be able to explore group dynamics and associations between interaction structures and outcomes [18–22]. Deeper understanding of interaction patterns in debriefings will contribute to more detailed knowledge of the effective underlying debriefing mechanisms and how communication between debriefers and participants influences simulation-based learning [6, 11, 23].

The purpose of the present study was to investigate possible different interaction patterns during debriefings, and the associations to short and after 1-month self-reported reactions, and learning outcomes using network analysis. We were interested if different interaction patterns influence participants’ learning outcomes. Specifically, this observational study aimed to find evidence about the following research questions:

1. How many distinguishable interaction patterns can be found?
2. How are the interaction patterns related to the subjective satisfaction of the debriefing by the participants? (Post-session survey)
3. How are the interaction patterns related to the perceived usefulness of the simulation sessions? (One-month follow-up)
4. How are the interaction patterns related to self-reported individual and team learning outcomes rated directly after simulation session? (Post-session survey)
5. How are the interaction patterns related to self-reported individual and team learning outcomes 1 month after the simulation session? (One-month follow-up).

## Methods

### Procedure

This observational study was performed at the departmental Bern Simulation and CPR-Centre at the Bern University Hospital in Bern, Switzerland, from January to December 2018. With informed consent from the simulation participants and instructors, twenty 5-h simulation sessions were observed live. Each 5-h simulation session comprised of an introduction (i.e. establish learning climate, clarifying the expectations and objectives), followed by three simulation scenarios, each with immediate debriefing after the scenario. The simulation sessions hosted each five to seven anaesthesia residents, specialists, and nurses. Immediately after the scenario, two certified simulation instructors (anaesthesia nurses and physicians) led the debriefings. All instructors included in the study have at least passed the EUSim simulation instructor course level 1. Scenarios plus debriefings were video recorded. There was no intervention in this observational study, but participants and debriefers were informed that interactions will be counted, and the simulation instructors were asked to debrief as per their usual practice (naturalistic approach).

In autumn 2017, prior to start of the study, two psychologists, who were trained to rate interactions, were introduced to the simulation environment and medical terminology. For each simulation session, they observed live both the simulation and the debriefing for all three scenarios. During each debriefing, they counted all speaking turns between participants and debriefers. These distinct interactions were noted in a ‘who-to-whom list’ [19, 21, 22].

### Surveys

Before the start of the first scenario, simulation participants filled in the first set of questionnaires, the second set was collected immediately after the debriefing of the third scenario to assess short-term self-reported learning outcomes, and the final set was collected 1 month later.

In the surveys, we first asked the participants about their demographics (i.e. age, gender, occupation, simulation experience). The presession survey set asked two questions rated on a visual analogue scale (VAS), ranging from 0 = very low to 10 = very high: (1) ‘How high is your actual motivation for today’s simulation?’ and (2) ‘How useful is today's simulation probably for your clinical work?’.

The post-session survey set was completed directly after the end of the 5-h simulation session and retrospectively assessed the following with a visual analogue scale (VAS), ranging from 0 = very low to 10 = very high: (1) the individual learning effects of each of the three debriefings (‘How much did you learn from the debriefing session for the first/second/third scenario?’), (2) the team learning effects of each of the three debriefings (‘How much do you think the team learnt from the debriefing session of the first/second/third scenario?’), (3) the satisfaction with the debriefings (‘How satisfied are you with the debriefing of the first/second/third scenario?’, and (4) the usefulness of the simulation session (‘How useful is today's simulation session for your clinical work?’). One month later, the participants received the same post-session survey set (follow-up).

### Establishing debriefing interaction patterns

**Fig. 1:**
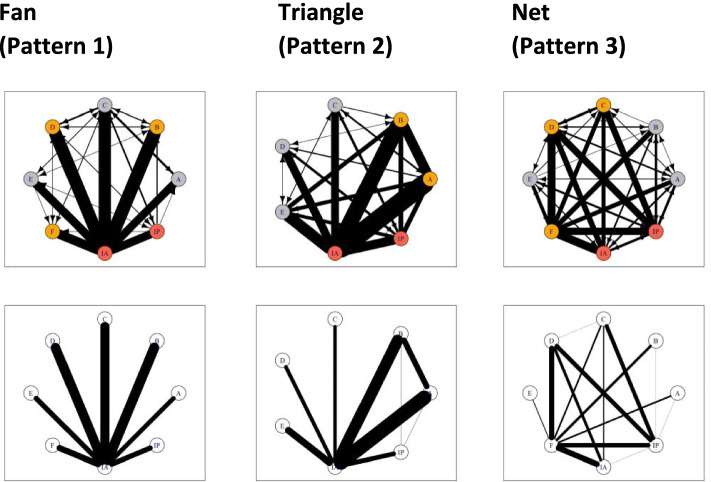
The three different interaction patterns for the debriefings. Top, full sociograms. Bottom, averaged sociograms from respective full sociograms. (All 57 interaction patterns are displayed in Additional file 3 (fan), Additional file 4 (triangle), and Additional file 5 (net)

Using the 57 ‘who-to-whom lists’ from the live observed debriefings, a qualitative-quantitative mixed network analysis was used to determine the number of distinctive interaction patterns. Based on these lists, we printed 57 network structures using the R-package *igraph *[24]. Three psychologists who were blinded to the research questions grouped these network structures into distinctive categories based on the printed averaged network structures, which are displayed at the bottom of Fig. 1. No constraints were placed on the number of categories to be used. After establishing interaction pattern categories, their discriminatory ability was evaluated by comparing seven common network metrics [25, 26] (Additional file 2). Network metrics refer to mathematical measures that use the underlying network matrix to capture specific properties of the network topology [16, 25, 26]. These metrics are used to validate the printed network structures and allow comparisons amongst networks of different sizes [25].

### Statistical analysis

Categorical data was analysed using Fisher’s exact tests and continuous variables by analysis of variance. Interrater agreement was assessed by Cohen’s kappa (κ) for categories and by intra-class correlations (ICC) for interval scaled data. A *p*-value of 0.05 or less was considered as statistically significant. The required sample size was estimated by prior power analysis using G*Power [27]. Assuming a medium effect size (*f*^2^ = 0.15) for linear regression (*α* = 0.05, 1-*β* = 0.80), the total required sample size was 55 debriefings.

We examined the relations between interaction pattern categories and learning outcomes using hierarchical linear multilevel regression analysis [28], which can be found in detail in Additional file 1.

All analyses were carried out using the R-package *nlme *[29] and *phia *[30] (De Rosario-Martinez 2015) in the R statistical language [31]. All of the models were estimated with maximum-likelihood estimation. Normal distribution of the outcome variables was confirmed by inspecting the residual diagnostics of the fitted multilevel models. We assessed the need for multilevel modelling by computing the respective intraclass correlation coefficients (ICCs).

### Missing data

In the post surveys, six responses of two different participants were missing (satisfaction ratings) and one for the usefulness ratings. To impute these missing values, we used the best-value regression algorithm from R-package *mice *[32].

## Results

Of the 21 simulation sessions, two sessions had to be excluded. In one session, three out of six participants did not consent; the other session was not ‘interprofessional’ as only physicians participated and no nurses, and therefore not comparable to the other debriefing teams. Thus, 19 simulation sessions, with 3 scenarios and debriefings each (total of 57 debriefings), were analysed. A total of 111 simulation participants, aged 39 ± 9 years, 60% female, were debriefed; 49% of them were nurses, 25% specialists, and 26% residents; all had participated in 2 ± 2 simulation sessions previously. More nurses than residents and specialists declined to answer the follow-up (*p* < 0.03). The overall response rate was 69%. Fourteen debriefers aged 43 ± 8 years, 21% female, with 5 ± 4 years of debriefing experience, formed 10 different pairs of debriefers.

Each debriefing lasted 36 ± 9 min and contained 625 ± 191 communication interactions. In total, 2076 min of debriefings was analysed, which contained 35,648 communication interactions. Interrater agreement was good to excellent (*ICCs* 0.78–0.99).

### Qualitative part: different interaction patterns during debriefings

Three blinded psychologists grouped the 57 printed network structures (Additional files 3, 4 and 5) into interaction patterns. Two raters defined three categories; one defined four. Based on the calculations of the interrater agreement, the fourth category was integrated into one of the other three, which provided acceptable to good interrater agreement (*κ* = 0.661 to *κ* = 0.879). Figure 1 displays the three different interaction patterns (all 57 interaction patterns are displayed in Additional files 3, 4 and 5):

- Pattern 1, the ‘fan’ is characterized by interaction between the debriefer and each individual participant.
- Pattern 2, ‘the triangle’ is characterized by interaction between the debriefer and each individual participant but also between two participants and the lead debriefer in a triangle shape.
- Pattern 3, ‘the net’ is characterized by interaction of the debriefer with each participant but also of interactions between all participants in a net formation.

In addition to a visual classification of network structures, social networks can be analysed using metrics that describe the density and shape in the network as well as the network centrality of individual members [25]. Discriminatory analysis of network metrics of the three interaction patterns revealed that pattern 1 (fan) is significantly different from pattern 3 (net) for all of the 10 evaluated network metrics (Additional file 2). Moreover, pattern 1 (fan) was significantly different from pattern 2 (triangle) in eight of 10 network metrics, and pattern 2 (triangle) differed significantly from pattern 3 (net) in one network metric.

### Quantitative part: results for satisfaction and usefulness

#### Intra-class correlation

Assessment of debriefing satisfaction as a function of the interaction pattern directly after the debriefings showed that 60.6% of the variance in subjective satisfaction was attributed to the participant level (i.e. nested in different simulation sessions). The ICC for measurement points nested in the participants was 0.253 and between the course groups 0.141. Perceived usefulness of the debriefings over time (pre, post, follow-up) as a function of the interaction pattern showed that 44.4% of the variance in usefulness was attributed to the measurement points. The ICC for participants nested in the simulation sessions was 0.377 and between the simulation sessions 0.180.

#### Associations with satisfaction

Table 1 shows the results for the final model of the multilevel regression analysis on all the short-term outcome variables. The only significant association was between participants’ satisfaction and motivation before the simulation session (*p* = 0.036). This suggests that the higher the motivation, the greater the satisfaction with the simulation session. There was no significant association between participants’ satisfaction and interaction pattern category, indicating no effect of interaction patterns on participants’ satisfaction ratings.

**Table 1:** Results of the multilevel regression analysis on the self-reported short-term outcome variables

Variables	Satisfaction		Individual learning		Team learning	
	***β***	***SE***	***β***	***SE***	***β***	***SE***
Intercept	0.001	0.103	0.000	0.073	0.001	0.076
Interaction category	0.007	0.041	0.184**	0.057	0.168*	0.067
Duration of debriefing	0.024	0.039	0.184*	0.057	0.120†	0.068
Time	0.023	0.034	0.081	0.051	0.053	0.062
Motivation	0.154*	0.082	0.260***	0.068	0.282***	0.054
Experience participants	− 0.146†	0.079	− 0.059	0.067	− 0.121*	0.051
Group size	0.084	0.113	− 0.046	0.082	0.015	0.085
Experience debriefers	0.238†	0.115	0.031	0.086	− 0.018	0.091
Pseudo *R*^2^ Nagelkerke^a^	0.106		0.206		0.166	
*χ*^2^-model test^b^	0.026		10.23**		6.08*	

^*^p < 0.05; **p < 0.01; ***p < 0.001; †*p* < 0.07

*Abbreviations*: *β* Standardized estimate, *SE* standard error

^a^Explained variance by the full model

^b^Chi-squared difference test between model 1 (covariates without pattern category) and model 2 (all predictors)

^c^Unstandardized variance

#### Associations with usefulness

Table 2 shows the results of the final model of the multilevel regression analysis on outcome variables after 1 month. Motivation was strongly positively associated with perceived usefulness over time (*p* < 0.001). There was no association for perceived usefulness of interaction pattern (*p* = 0.259) or for the interaction of interaction pattern with time (*p* = 0.112). This indicates no effect of interaction patterns on participants’ perceived usefulness of the whole simulation session.

**Table 2:** Results of the multilevel regression analysis on self-reported outcome variables after 1 month

Variables	Usefulness		Individual learning		Team learning	
	***β***	***SE***	***β***	***SE***	***β***	***SE***
Intercept	0.024	0.074	− 0.020	0.0835	− 0.019	0.084
Time	0.026	0.041	− 0.145*	0.056	− 0.149**	0.054
Motivation	0.532***	0.060	0.378***	0.076	0.374***	0.076
Experience participants	− 0.021	0.059	− 0.010	0.078	− 0.042	0.078
Interaction category	0.123	0.102	0.109	0.114	0.111	0.115
Group size	0.018	0.081	0.022	0.090	0.048	0.091
Experience debriefers	− 0.056	0.101	− 0.016	0.120	− 0.047	0.120
Interaction category × time	0.066	0.042	0.064	0.057	0.089	0.055
	**Estimate**		**Estimate**		**Estimate**	
Pseudo *R*^2^ Nagelkerke^a^	0.323		0.251		0.269	
*χ*^2^-model test^b^	3.737		1.947		3.277	

^*^p < 0.05; **p < 0.01; ***p < 0.001; †*p* < 0.07

*Abbreviations*: *β*, standardized estimate; *SE*, standard error

^a^Explained variance by the full model (with all predictors)

^b^Chi-squared difference test between model 1 (covariates without pattern category) and model 2 (all predictors)

^c^Unstandardized variance

### Results for self-reported learning outcomes

#### Intra-class correlation

Post-simulation session assessment of individual learning as a function of the interaction pattern showed that 64.0% of the variance in the individual learning was attributed to the measurement points nested in the participants (level 1). The ICCs for variability in the short-term individual learning for participants nested in the simulation sessions 0.303 and between the simulation sessions 0.057. Post-simulation session assessment of team learning showed the ICC for measurement points nested in participants was 0.497; for the participants nested in the simulation sessions, 0.457; and between the simulation sessions, 0.046.

The 1-month follow-up revealed that 57% of the variance in the individual learning was attributed to the measurement points (level 1). The ICC for variability in individual learning for participants nested in the simulation sessions was 0.335 and between the simulation sessions 0.095. For 1-month follow-up team learning, the ICC for measurement points nested in the participants was 0.554; for the participants nested in the simulation sessions, 0.351; and between the simulation sessions, 0.095.

#### Associations with short-term individual and team learning

**Fig. 2:**
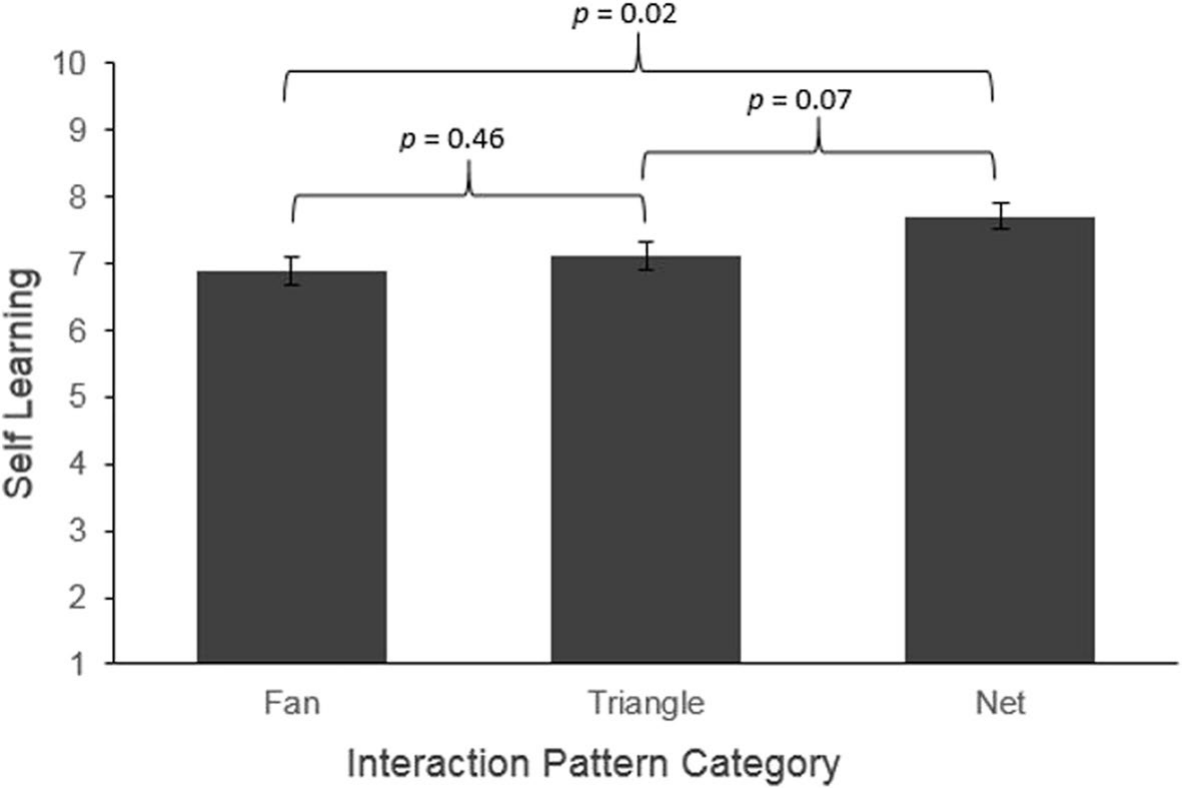
Simple effects of interaction pattern on self-learning

**Fig. 3:**
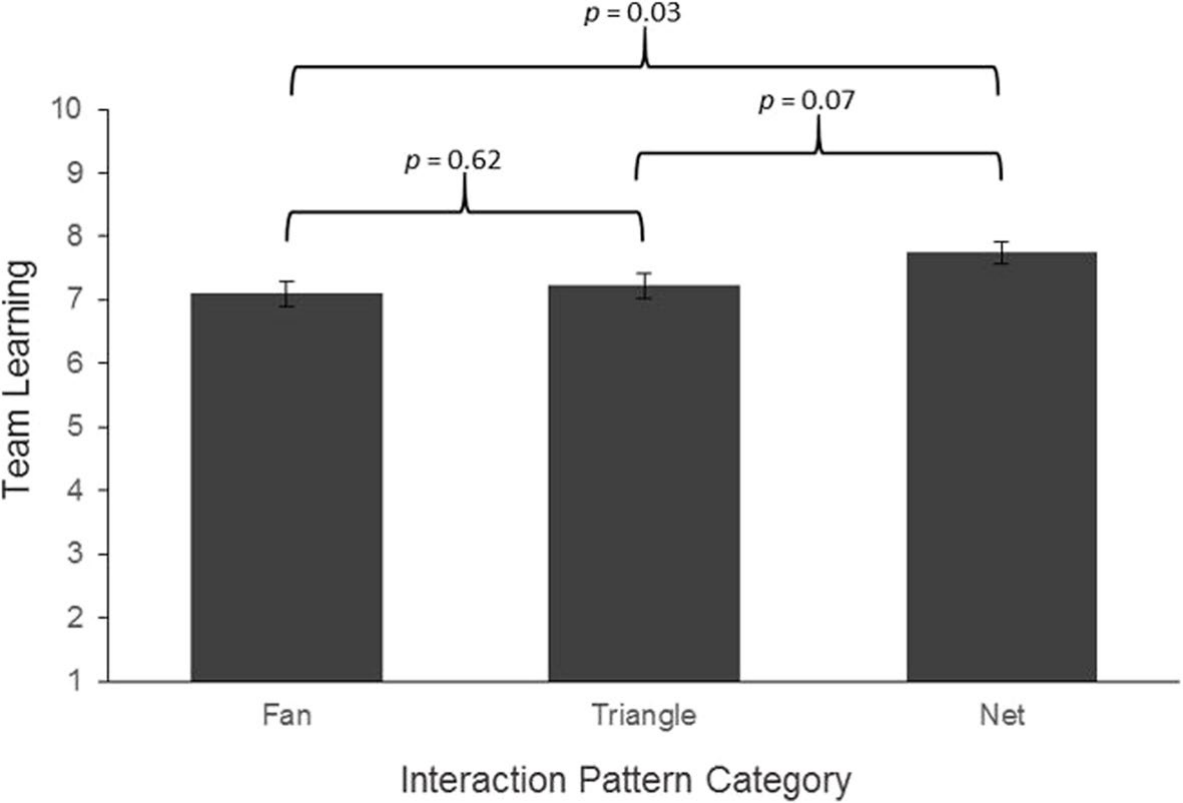
Simple effects of interaction pattern on team learning

The results showed nonsignificant random effects for individual learning and team learning (Table 1). However, the interaction pattern had a significant effect on individual learning (*p* = 0.002) and team learning (*p* = 0.017). Post hoc* contrast analysis* revealed a significant mean difference for individual learning between pattern 1 (fan, 6.89 ± 0.22) and pattern 3 (net, 7.71 ± 0.20; *p* = 0.016) (Fig. 2). Similarly, a significant difference for team learning was seen between pattern 1 (fan, 7.10 ± 0.19) and pattern 3 (net, 7.74 ± 0.18; *p* = 0.03) (Fig. 3). Thus, the learning effects were significantly higher in the debriefings with a net pattern compared to those with a fan pattern. All other pattern comparisons were not significant.

Motivation was significantly associated to individual and team learning as assessed directly post-simulation (*p* < 0.001). This suggests that the higher the motivation, the greater the perceived learning effects. Also, simulation experience was significantly negatively associated to team learning (*p* = 0.018), but not to individual learning (*p* = 0.378). This indicates that greater prior experience with simulations was associated with significantly smaller ratings of a team learning effect. Longer duration of debriefings was significantly positively associated with individual learning (*p* = 0.001), but not to team learning (*p* = 0.086), suggesting that longer debriefings yielded higher ratings on individual learning.

#### Associations with individual and team learning after 1 month

Table 2 shows the results of the final model of the multilevel regression analysis of 1-month follow-up. There was a significant effect of time on individual and team learning. Learning decreased over time, which was more pronounced for team learning. Motivation before the simulation session was strong positively associated with individual and team learning (*p* < 0.001), which suggested a strong and lasting effect of participant motivation on learning outcomes. There was no effect of interaction pattern on individual or team learning (*p* = 0.354; *p* = 0.424, respectively), and no interaction with time for either of these learning outcomes (*p* = 0.270; *p* = 0.112, respectively).

## Discussion

This study revealed three interrelated main findings that might enhance debriefing quality. First, there were three interaction pattern categories (fan, triangle, and net; Fig. 1) distinguishable by network metrics [25, 26] (Additional file 2). Second, these interaction patterns were associated with self-reported short-term learning effects, and third, participants’ motivation before the simulation session was highly predictive for self-reported learning, satisfaction, and usefulness.

Interestingly, two previously described patterns [6] emerged (i.e. fan, triangle). Additionally, we found the net structure, which has a lot of similarities to the previously described star pattern by Dieckmann and collegues [6]. Contrary to the star pattern, the net structure shows strong interactions between all participants. This pattern significantly enhances self-reported short-term individual and team learning. The fan pattern represents the earlier described instructor-centred debriefing style, where most of the communication activity is directed by the debriefer, and no interactions between participants occur [11, 33–35]. The triangle pattern is characterized by interactions of the lead debriefer with two of the most active participants, which also includes some interactions between these two participants [6]. The net pattern relates to what is called a learner-centred debriefing style [11, 33–35], in which the communication is mostly balanced between all of the group members and the debriefers. Our findings therefore support the assumption of common interaction patterns in debriefings of simulations [6].

There was a significant association of the net interaction patterns on self-reported short-term learning effects. Participants who experienced the net interaction pattern reported significantly greater short-term learning effects in individual and team learning compared to the fan interaction pattern. These findings are in line with current good-practice debriefing guidelines [8, 10, 12, 13] and the growing evidence on learner-centred approaches [11, 33–35]. That is, participants were actively engaged in debriefings under the facilitation of a well-trained debriefer who intended to maximize the learners’ development [10, 11, 33, 34], and that active engagement of learners in knowledge construction is essential in the process of learning [11, 33–35]. Based on our results, we argue that debriefers might operate as facilitators of the group’s discussion [6, 8, 12] and should encourage participants to exchange their own reflections, regardless of their role in the simulation scenario [6, 8]. Thus, effective debriefing occurs when the debriefers focus not only on the content of the debriefing explicitly but also on the process and structure of the discussion (i.e. managing: transmission from one topic to the next, time, balancing participant contributions) [12].

In contrast to this, instructor-centred teaching implies unilateral control of the learning content and time spent on each issue, with a disparity of power [11]. Such behaviour might jeopardize the psychological safety of participants and burdens the responsibility for learning on the debriefers [11]. Thus, participants might show less self-regulation, less self-assessment, and fail to identify performance gaps [11]. Nevertheless, instructor-centred debriefing might be appropriate for different target groups and topics. For instance, a recent study showed that local culture is related to debriefing practices and perceived engagement of the participants (i.e. hierarchy) [36]. Open questions therefore are as follows: the implication of debriefing styles in interprofessional cultures, in interdisciplinary cultures, and with participants of different educational levels (i.e. students, postgraduate learners, specialists).

Interestingly, the different interaction patterns had no significant effects on the self-reported learning effects assessed 1 month after the simulation session. Generally, learned competences decrease over time [37]. In the present study, the time effect and motivation mainly explained the variance in the model (Table 2). Nevertheless, post hoc analysis of this nonsignificant effect showed that the self-reported learning effects of participants who experienced the net interaction patterns remained relatively stable, whereas it decreased over time in the other interaction patterns. Also, the interaction patterns showed neither significant associations with participants’ satisfaction with debriefings nor on the perceived usefulness. Other open questions therefore are the unclear association of the complex relationship between interaction pattern and participant satisfaction as well as usefulness of simulation.

Finally, participant motivation prior to the start of simulation was highly predictive for significantly higher rates in usefulness, satisfaction, and self-reported learning effects directly after the simulation session (Table 1) and after 1 month (Table 2). There is strong evidence that motivation is related to skill acquisition, willingness to learn, and implementation of newly acquired skills into practice [35]. It seems possible that participants’ motivation could influence the debriefing interactions and therefore the interaction patterns. However, using the methodology of this study, this question cannot be reliably answered but should clearly be addressed in future debriefing research.

### Strengths and limitations

A strength of our study is the high number of 57 debriefings, which were coded by two trained raters and showed a high interrater reliability. In contrast to a previous observational study with a single rater for interaction patterns based on eight different debriefings [6], the present study used a more rigorous methodological and statistical approach. Furthermore, we applied mixed quantitative–qualitative network methodology [14, 17–19, 22] and evaluated pattern distinctiveness using established network metrics [25, 26]. We controlled different systems and participant variables in our models. For instance, research has shown that group size influences the distribution of participation in teams [21, 22, 38]. Furthermore, we included the number of participants per debriefing group as a control variable in our statistical models. Even after controlling for these variables, our results remained statistically significant.

Our study has several limitations. As the authentic debriefing sessions were video recorded, the presence of a video camera might had an effect on participants’ and debriefers’ behaviour in the debriefings. However, it has been shown that using video recording has no serious influence on participant behaviour [19, 21].

We aggregated all of the interactions into a whole interaction pattern during the entire debriefing, developing our patterns, which were therefore static. Thus, we cannot make statements about the optimal interaction dynamics during the debriefings. It is possible that a more directive debriefing style was constructive during some of the debriefing phases [12]. For example, a more instructor-centred debriefing style might also be beneficial when establishing psychological safety, setting the agenda, and clarifying the expectations [12, 14, 19].

All of the debriefings were collected in one simulation centre. The results might differ in different cultures or with different target groups[36]. Therefore, to strengthen the generalizability, further studies in different settings should be performed.

Finally, we assessed each learning outcome by one self-reported item. This might influence the validity of our results, because of a possible bias (i.e. social desirability, acquiescent bias). The accuracy of self-reported learning outcomes has been the focus of intense debate [39–41]. Self-assessment of learning is sometimes regarded as having dubious validity compared to so-called direct measures of learning [42] (i.e. observer ratings, clinical outcomes). Self-reported learning measurements are only indirect indicators of increased learning and have been shown to be biased [39, 40]. Nevertheless, studies have shown that self-assessment might be appropriate in scholarly research especially for high-performing persons [40, 42, 43] and reported correlations of self-reported learning and external final exam scores as moderate to high [39, 41]. Although evidence is mixed about the validity of self-reported learning outcomes [39, 41], and considering the weaknesses and strengths of this type of measurement, we argue that self-reported learning outcomes are appropriate with well-experienced anaesthesiologists and nurses used to regularly scheduled simulation sessions and may contribute to a better understanding of learning processes in debriefings. Nevertheless, future research should incorporate a more advanced learning outcome measure to capture individual learning effects.

## Conclusion

This study revealed three different interaction patterns during debriefings: fan, triangle, and net. As a particular empirical investigated novelty, the net pattern was significantly associated with improved self-reported short-term individual and team learning. These results are in line with current good practice debriefing guidelines and the growing evidence on learner-centred debriefings. The practical implications are that knowledge of different debriefing styles enhances the debriefers’ ability to act and opens the possibility to stimulate active engagement of learners. Focusing additionally on the debriefing process, rather than only on the content of the debriefing, may influence short-term learning and possibly enhances the efficacy of simulation-based medical education. Motivation before simulation sessions has a strong influence on self-reported learning outcomes and should be enhanced.

## Supplementary Information

**Additional file 1. **Statistical Analyses – Model Structure and Specification.

**Additional file 2. **Network metrics and dyad census for the different interaction patterns.

**Additional file 3. **All network structures Pattern 1: Fan.

**Additional file 4. **All network structures Pattern 2: Triangle.

**Additional file 5. **All network structures Pattern 3: Net.

## Data Availability

Restrictions apply to the availability of these data, and so, they are not publicly available. Data are available from the corresponding author upon reasonable request and with the permission of the Bern Ethics Committee, according to the Swiss Federal Human Research Act.
