# Supplementary material for: Debriefing interaction patterns and learning outcomes in simulation: an observational mixed-methods network study
Source: Adv Simul (Lond). 2022 Sep 6;7:28. doi: 10.1186/s41077-022-00222-3 (PMC9450386; doi:10.1186/s41077-022-00222-3)
Supplement: Supplementary file 1 — Additional file 1. Statistical Analyses – Model Structure and Specification. [file 41077_2022_222_MOESM1_ESM.docx]

**Additional File 1**

**Statistical Analyses – Model Structure and Specification**

The hierarchical structure of the data (i.e., participants nested in simulation session groups across survey sets) required hierarchical linear multilevel regression analysis^28^, which allowed to control for effects on the debriefing group (i.e. different debriefing styles experienced). For all models, we specified the intercepts as randomly varying among participants within simulation sessions, with the slopes fixed within each simulation session.

Interaction patterns in relation to subjective satisfaction with the debriefing and short term self-reported learning outcomes were addressed by a three-level model: Level I: three debriefings (first, second, third) were nested in the participants (Level II) nested in the simulation sessions (Level III).

Interaction pattern in relation to self-reported learning outcome 1-month after debriefing was addressed by a three-level-model with survey sets (Level I) nested in the participants (Level II) nested in the simulation sessions (Level III).

Interaction pattern in relation to perceived usefulness of the simulation session 1-month after simulation sessions was modelled by a three-level model, where all three surveys (Level I) were nested in the participants (Level II) nested in the simulation sessions (Level III). As we assessed usefulness only for the whole simulation session, we coded the dominant interaction pattern across all three debriefings of one simulation session, as: 1, fan was dominant; 2, triangle was dominant; 3, net was dominant; and 0, variable (three different interaction patterns present).

For all these models, we specified the intercepts as randomly varying among participants within simulation sessions, with the slopes fixed within each simulation session.
