# Supplementary material for: Debriefing interaction patterns and learning outcomes in simulation: an observational mixed-methods network study
Source: Adv Simul (Lond). 2022 Sep 6;7:28. doi: 10.1186/s41077-022-00222-3 (PMC9450386; doi:10.1186/s41077-022-00222-3)
Supplement: Supplementary file 2 — Additional file 2. Network metrics and dyad census for the different interaction patterns. [file 41077_2022_222_MOESM2_ESM.docx]

**Additional file 2:** Network metrics and dyad census for the different interaction patterns.

| **Network metric** | **Pattern 1: Fan** | | **Pattern 2: Triangle** | | **Pattern 3: Net** | |  | |
| --- | --- | --- | --- | --- | --- | --- | --- | --- |
|  | (*n* = 18) | | (*n* = 18) | | (*n* = 21) | |  | |
|  | ***M*** | ***SD*** | ***M*** | ***SD*** | ***M*** | ***SD*** | ***F-test*** | **ɳ^2a^** |
| Density | 0.657 | 0.136 | 0.782 | 0.957 | 0.814 | 0.105 | 10.70*** | 0.27 |
| Centralization | 0.303 | 0.117 | 0.197 | 0.083 | 0.174 | 0.094 | 9.21*** | 0.25 |
| Clustering | 0.742 | 0.129 | 0.856 | 0.073 | 0.891 | 0.065 | 13.39*** | 0.33 |
| Hierarchy | 0.222 | .108 | 0.185 | 0.102 | 0.164 | 0.079 | 1.76 | 0.06 |
| Diameter | 25.39 | 11.54 | 15.61 | 4.00 | 13.24 | 4.62 | 14.22*** | 0.35 |
| Average strength R | 412.84 | 120.91 | 308.07 | 95.13 | 246.48 | 82.55 | 13.59*** | 0.36 |
| Average strength N | 70.20 | 57.40 | 120.72 | 52.52 | 194.53 | 74.27 | 19.39*** | 0.42 |
| Dyads mutual | 14.78 | 3.63 | 18.11 | 3.50 | 20.43 | 3.50 | 12.37*** | 0.31 |
| Dyads asymmetric | 6.06 | 3.06 | 5.00 | 2.99 | 4.57 | 2.16 | 1.48 | 0.05 |
| Dyads null | 6.44 | 4.33 | 3.39 | 2.28 | 3.05 | 3.04 | 5.95** | 0.18 |

*, *p* <0.05; **, *p* <0.01; ***, *p* <0.001

Abbreviations: M, mean; SD, standard deviation; R, resident; N, nurse

^a^ Effect size eta squared

**Glossary Terms**

A network is a system whose elements are connected in some way. The elements of a system are represented as nodes and the connections between interacting elements are called edges or links^19^.

- Density = Provides information about the degree of connectedness of the nodes of a network. Density is calculated as the ratio of existing relationships between nodes to the maximum number of possible relationships of nodes in the network. Possible value between 0 (=no relationships (links) between participants (nodes) and 1 (= maximum possible number of relationships (links) between participants (nodes))^24^
- Centralization = Centralization of a network is a measure of how central its most central node is in relation to how central all the other nodes are. It is calculated as the sum in differences in centrality between the most central node in a network and all other nodes. Possible values between 0 (= no singular authority) and 1 (= a singular authority retains total control over all aspects of the network)^24^
- Clustering = networks’ tendency to form well-connected sub-groups. It is interpreted as the tendency for sub-groups to form more internal ties and be linked to other groups via fewer ties. Possible value between 0 (=no subgroups) and 1 (= maximum possible subgroups)^24^
- Hierarchy = Hierarchy describes how frequent redundant relationships between nodes are and how strong information flows from the top level of the network down. Possible values between 0 (= many redundant relationships) and 1 (= no redundant relationships)^24^
- Diameter = The diameter of a network is the maximum geodesic distance within a network If the length of the path, measured by the number of connecting edges between any two nodes, is called the distance between them, then the geodesic distance is the shortest path between them. The higher the value, the broader the network.^19^
- Average strength = It is the sum of the weights (in this study the number of interactions) of edges connected to the node. The higher the value, the more the resident (R) or the nurse (N) interacted with other participants.^19^
- Dyads = A dyad consists of an unordered pair of nodes and links that exist between two actors of the pair. The dyadic relation between any two actors in a given network must have one of the three possible states: mutual (reciprocal), asymmetric (only one node interacted with another node) or null (neither node has a link to the other). The higher the number the more dyads are present in the network.^25^
