# Supplementary figures and images for: Debriefing interaction patterns and learning outcomes in simulation: an observational mixed-methods network study
Source: Adv Simul (Lond). 2022 Sep 6;7:28. doi: 10.1186/s41077-022-00222-3 (PMC9450386; doi:10.1186/s41077-022-00222-3)

**Additional File 3:** All network structures Pattern 1: Fan.

*
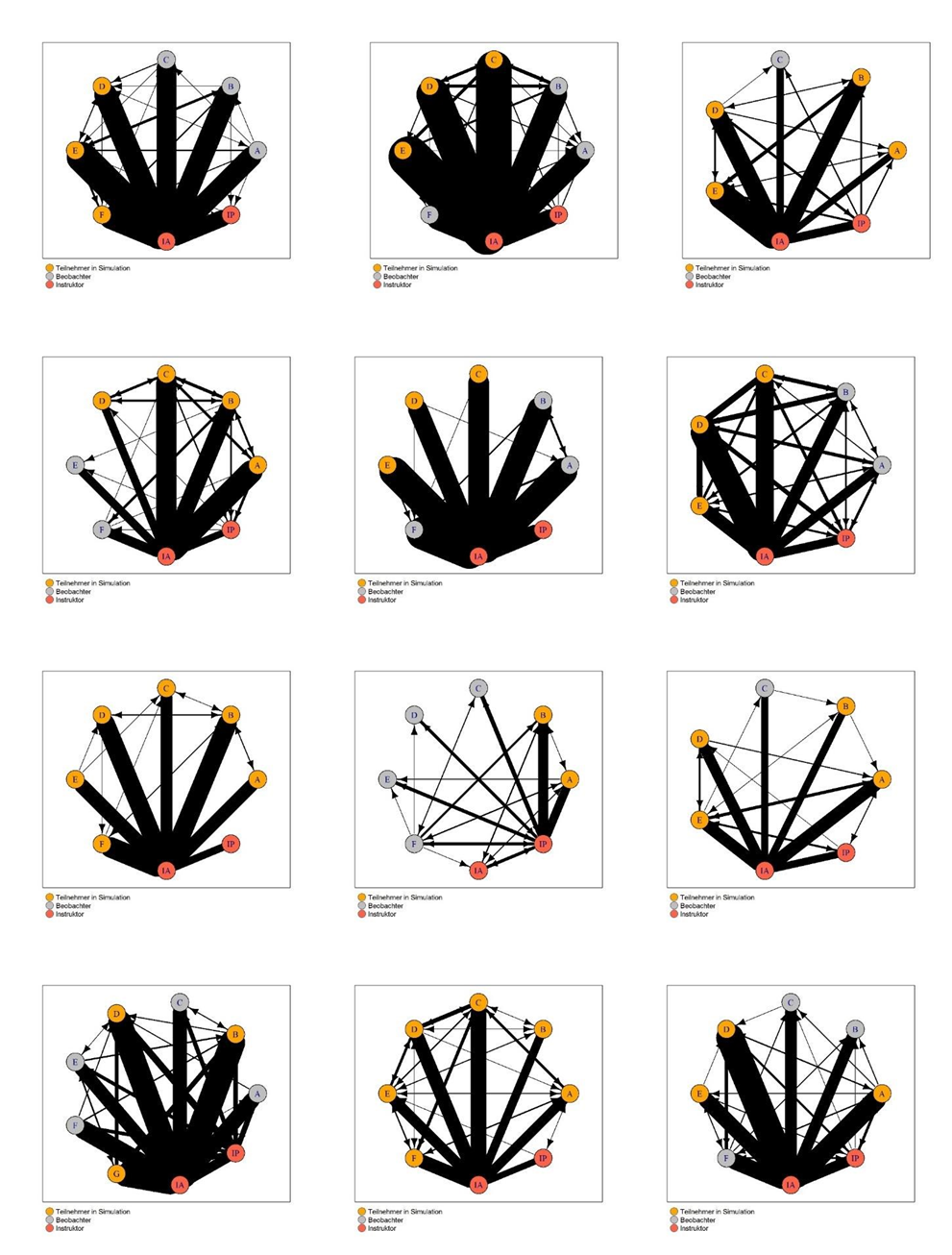
*

*
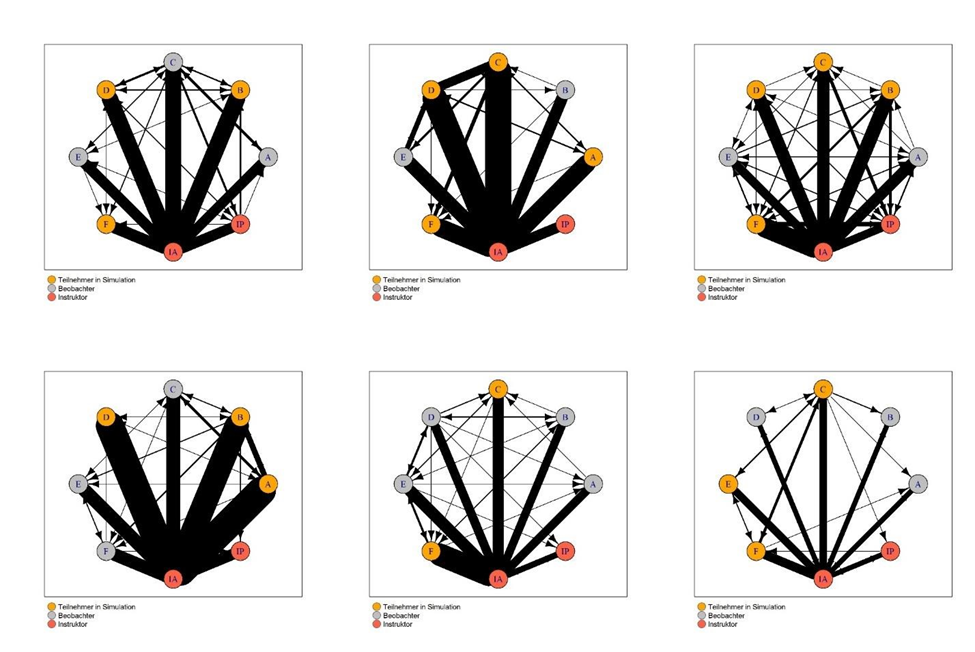
*

Supplement: Supplementary file 3 — Additional file 3. All network structures Pattern 1: Fan. [file 41077_2022_222_MOESM3_ESM.docx]

**Appendix 4:** All network structures Pattern 2: Triangle.


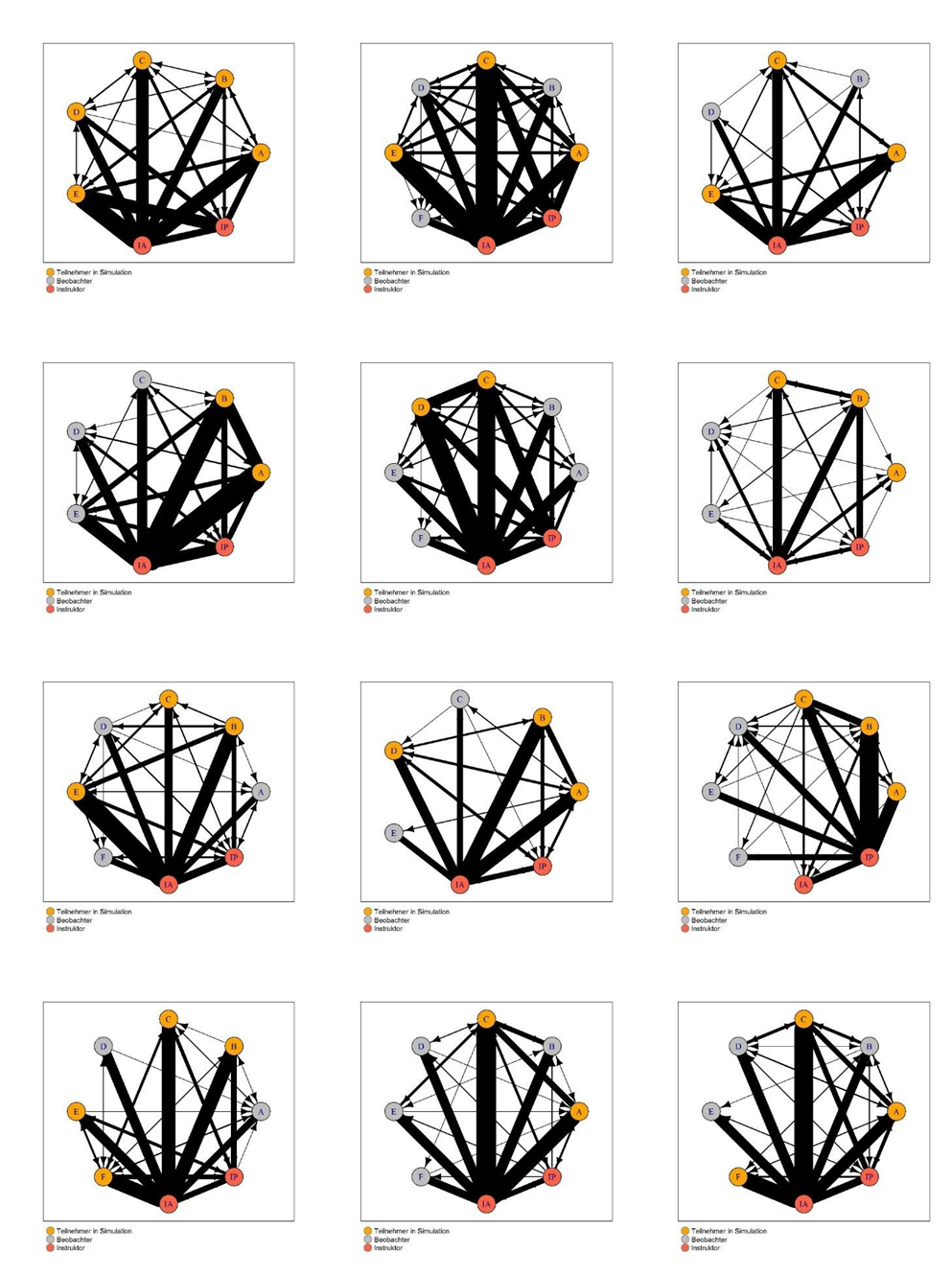


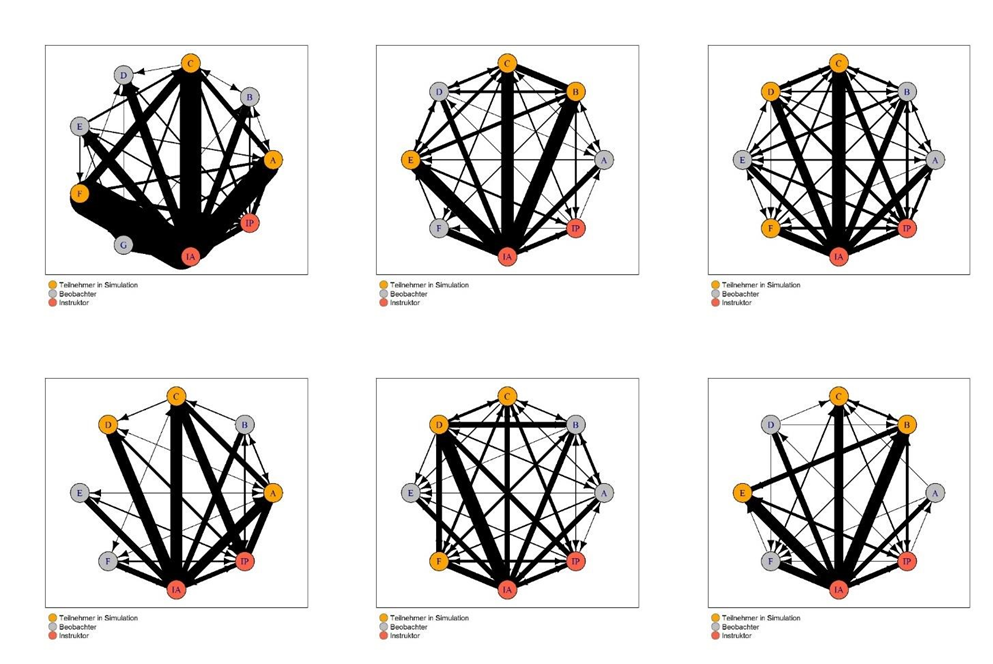

Supplement: Supplementary file 4 — Additional file 4. All network structures Pattern 2: Triangle. [file 41077_2022_222_MOESM4_ESM.docx]

**Appendix 5:** All network structures Pattern 3: Net.


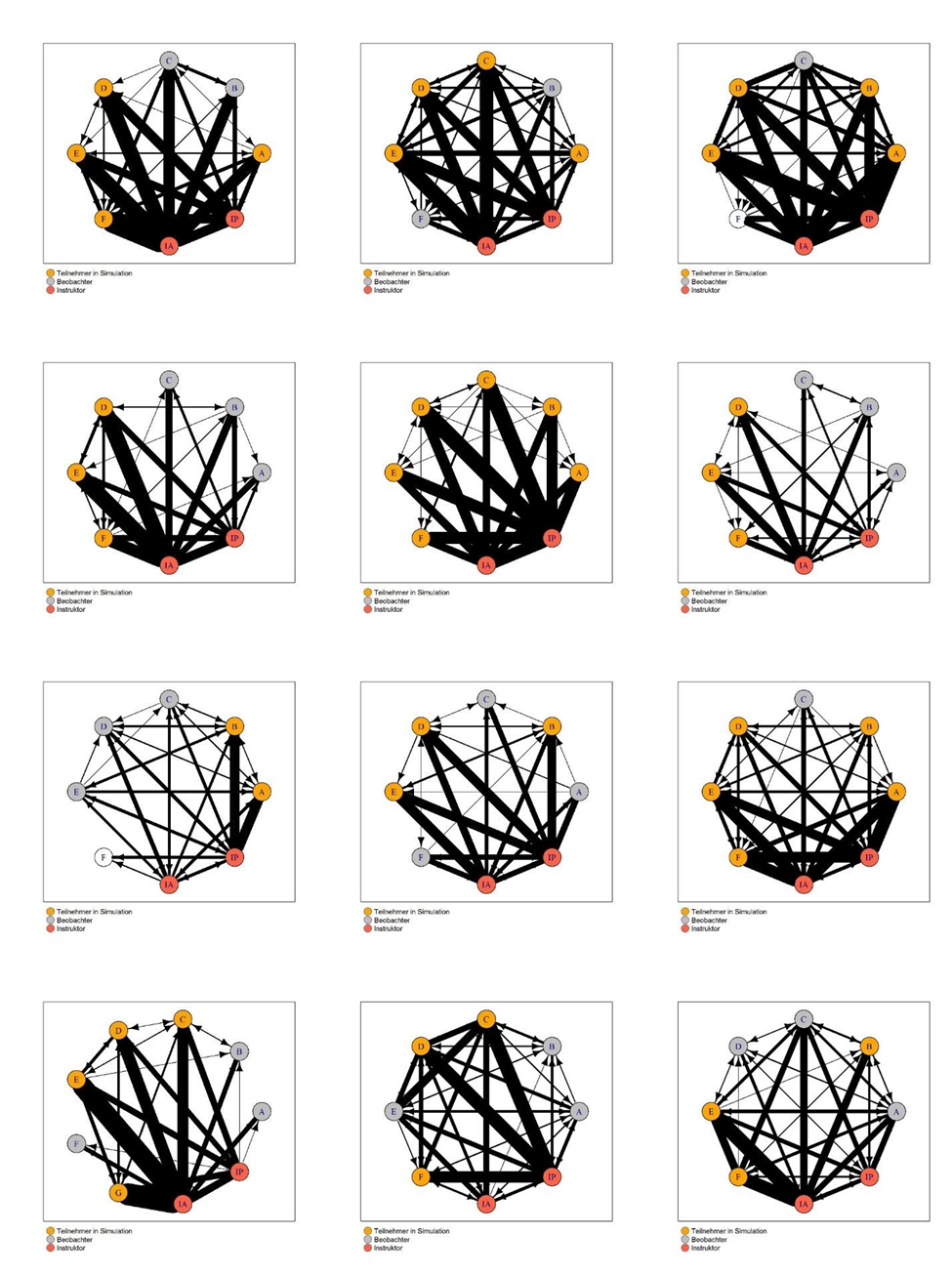


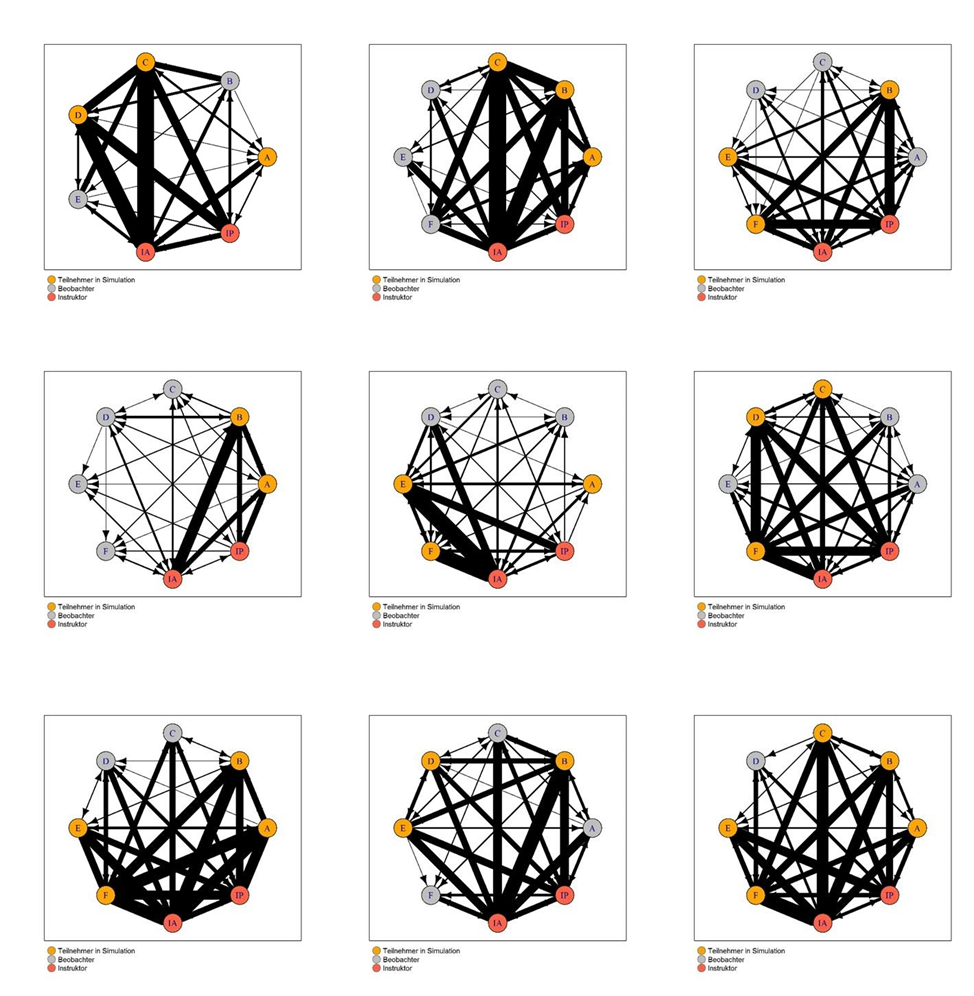

Supplement: Supplementary file 5 — Additional file 5. All network structures Pattern 3: Net. [file 41077_2022_222_MOESM5_ESM.docx]
